# Endoplasmic Reticulum Stress and Autophagy Markers in Soleus Muscle Disuse-Induced Atrophy of Rats Treated with Fish Oil

**DOI:** 10.3390/nu13072298

**Published:** 2021-07-03

**Authors:** Gabriel Nasri Marzuca-Nassr, Wilson Mitsuo Tatagiba Kuwabara, Kaio Fernando Vitzel, Gilson Masahiro Murata, Rosângela Pavan Torres, Jorge Mancini-Filho, Tatiana Carolina Alba-Loureiro, Rui Curi

**Affiliations:** 1 Department of Internal Medicine, Faculty of Medicine, Universidad de La Frontera, Temuco 4780000, Chile; 2 Department of Physiology and Biophysics, Institute of Biomedical Sciences, University of São Paulo, São Paulo 05508-000, Brazil; mit_macae@yahoo.com.br (W.M.T.K.); K.Vitzel@massey.ac.nz (K.F.V.); taticaloureiro@gmail.com (T.C.A.-L.); ruicuri59@gmail.com (R.C.); 3 School of Health Sciences, College of Health, Massey University, Auckland 0745, New Zealand; 4 Nephrology Division, Medical Investigation Laboratory-29 (LIM-29), Medical School, University of São Paulo (FM-USP), São Paulo 01246-903, Brazil; gilmasa@gmail.com; 5 Department of Lipids Laboratory, Food Science & Nutrition, Faculty of Pharmaceutical Science, University of São Paulo, São Paulo 05508-000, Brazil; rptorres@usp.br (R.P.T.); jmancini@usp.br (J.M.-F.); 6 Interdisciplinary Post-Graduate Program in Health Sciences, Cruzeiro do Sul University, São Paulo 01506-000, Brazil; 7 Butantan Institute, São Paulo 05508-040, Brazil

**Keywords:** ω-3 fatty acids, eicosapentanoic acid, docosahexaenoic acid, hindlimb suspension, skeletal muscle atrophy, unfolded protein response

## Abstract

Endoplasmic reticulum stress (ERS) and autophagy pathways are implicated in disuse muscle atrophy. The effects of high eicosapentaenoic (EPA) or high docosahexaenoic (DHA) fish oils on soleus muscle ERS and autophagy markers were investigated in a rat hindlimb suspension (HS) atrophy model. Adult Wistar male rats received daily by gavage supplementation (0.3 mL per 100 g b.w.) of mineral oil or high EPA or high DHA fish oils (FOs) for two weeks. Afterward, the rats were subjected to HS and the respective treatments concomitantly for an additional two-week period. After four weeks, we evaluated ERS and autophagy markers in the soleus muscle. Results were analyzed using two-way analysis of variance (ANOVA) and Bonferroni post hoc test. Gastrocnemius muscle ω-6/ω-3 fatty acids (FAs) ratio was decreased by both FOs indicating the tissue incorporation of omega-3 fatty acids. HS altered (*p* < 0.05) the protein content (decreasing total p38 and BiP and increasing p-JNK2/total JNK2 ratio, and caspase 3) and gene expressions (decreasing BiP and increasing IRE1 and PERK) of ERS and autophagy (decreasing Beclin and increasing LC3 and ATG14) markers in soleus. Both FOs attenuated (*p* < 0.05) the increase in PERK and ATG14 expressions induced by HS. Thus, both FOs could potentially attenuate ERS and autophagy in skeletal muscles undergoing atrophy.

## 1. Introduction

Aging, catabolic states (e.g., cancer and sepsis), and lack of mechanical load cause skeletal muscle atrophy. There are several strategies to treat skeletal muscle atrophy [1,2,3,4]. One of them is dietary supplementation [5,6], including administration of fish oils (FOs) [7]. FOs contain high amounts of eicosapentaenoic (EPA) and docosahexaenoic (DHA), ω-3 fatty acids (FAs). Previous studies reported that these FAs improve conditions with marked skeletal muscle mass loss [8,9,10]. Administration of ω-3 FAs increases protein synthesis [11,12,13] and decreases protein degradation signaling [14,15] in the skeletal muscle. 

Endoplasmic reticulum stress (ERS) promotes unfolded protein response (UPR), reducing protein synthesis and augmenting the content of chaperones and of protein degradation and autophagy markers. ERS also impacts muscle metabolism, adaptation, and remodeling [16,17]. 

UPR has three signaling branches: UPR activates inositol requiring kinase 1 (IRE1), double-stranded RNA-activated protein kinase-like endoplasmic reticulum kinase (PERK) and activating transcription factor 6 (ATF6) [18]. IRE1α cleaves X-box binding protein (XBP1) mRNA removing 26 nucleotides and generating the spliced XBP1 (sXBP1) that promotes transcription of UPR genes. Some IRE1/XBP1 via activated genes takes part in endoplasmic reticulum (ER)-associated degradation signaling [18]. IRE1α-mediated XBP1 mRNA processing promotes rapid breakdown and elimination of rough ER-associated mRNAs, decreasing entry of new proteins into this organelle lumen. When there is a chronic stress condition, activated IRE1α can trigger downstream phosphorylation of the pro-apoptotic kinase jun N-terminal kinase (JNK) or p38 members of MAP kinase (MAPK) family [19,20].

Activated PERK raises transcription of UPR regulation genes (through PERK-eIF2α-ATF4 pathway), like chaperones binding immunoglobulin protein (BiP) among others [21,22]. These genes are associated with oxidative stress suppression and stimulation of autophagy and cell metabolism [23].

The three signaling branches of UPR promote cell apoptosis through CCAAT/enhancer-binding protein homologous protein (CHOP) activation [20,24]. CHOP also regulates apoptosis directly or indirectly affecting the activity of caspase 3 [20]. In addition to mediating apoptosis through the endogenous and exogenous pathways, CHOP also mediates apoptosis through other pathways indirectly increasing the oxidation of protein disulfide isomerase (PDI) [20,25]. 

UPR activation is also associated with increased autophagy activity [26]. Autophagy is an evolutionarily well-preserved degradation process that maintains cellular homeostasis and responds to various cellular stressors. It can selectively or non-selectively degrade cellular components. A gain or a loss of autophagic function can induce cell damage [27,28,29].

Autophagy is essential for preventing accumulation of misfolded/aggregated proteins and controlling muscle glucose levels, energy balance, and exercise response. In opposition, increased autophagy activity lowers muscle protein synthesis [30,31,32]. Due to these observations, there is an urgent need to investigate autophagy process during skeletal muscle-induced atrophy conditions. In this line, Beclin, LC3b, and ATG14, markers of the macroautophagy, are important to consider [33], which involves 4 phases: initiation, formation, membrane expansion, and maturation [34,35].

Deldicque and colleagues (2010) reported that high-fat diet (HFD) fed mice exhibit augmented BiP and IRE1α content in soleus muscle. These authors also reported UPR activation in response to palmitic acid in C2C12 myogenic cells [36]. As mentioned above, stressful conditions activate the soleus UPR, which is the case of long-distance running [37]. On the other side, bed rest for nine days also activates UPR in human muscle [38]. The above, increasing the activity of the 26S proteasome or the autophagy process, leads to skeletal muscle atrophy [39]. 

Using a hindlimb suspension (HS)-animal model some authors observed an increase in UPR [40] and autophagy [41] markers whereas others did not [42]. ω-3 FAs have had different effects on these markers. For example, EPA attenuated lipotoxicity-induced cardiomyocyte apoptosis through autophagy regulation [43] and DHA modulated ubiquitin proteasome and autophagy indicating that it could delay muscle mass loss due to aging [44]. In addition, DHA supplementation prior to fasting preserved muscle mass by reducing activation of AMPK, ubiquitination or autophagy [10]. On the other hand, ω-3 fatty acids supplementation increases muscle atrophy caused by glucocorticoids in an autophagic, AMPK and ubiquitination process [45]. In this sense, understanding the effects of two FOs supplementation in a skeletal muscle disuse condition on UPR and autophagy markers might shed light on novel therapies for skeletal muscle atrophy.

Along these lines, skeletal muscle atrophy involves an inflammatory phase that occurs during the first 3–5 days of the process, leading to cell death and tissue remodeling with an activation of ERS and autophagy [46,47,48,49]. ERS activates UPR proteins, which trigger a sterile inflammation [50]. Herein, we investigated the effect of anti-inflammatory EPA or DHA-rich FOs [51] on UPR and autophagy in muscle atrophy induced by HS. It was hypothesized that this treatment might decrease UPR and autophagy markers activity in soleus muscle undergoing a disuse-induced atrophy. 

This study comprises of a large research project investigating ω-3 FAs (high EPA and high DHA) in skeletal muscle atrophy induced by HS; we previously published results on protein synthesis/protein degradation signaling showing that this experimental model promotes a reduction of body weight, fat mass, muscle weight, and soleus cross-sectional areas (CSA), and a decrease in protein synthesis and an increase protein degradation [7]. FOs supplementations did not influence changes in body weight or soleus CSA induced by HS. However, in the percentage of soleus fibers CSA from rats submitted to HS, there was an increase in the number of fibers in the range of 1000 µm^2^ by approximately 140%. On the other hand, with respect to fibers in the range of 800 µm^2^, supplementation with both fish oils during HS caused a reduction of about 25% compared with controls. EPA-high fish oil attenuated the changes induced by HS on 26S proteasome activity, and levels of p-Akt, total p70S6K, p-p70S6K/total p70S6K, p-4EBP1, p-GSK3-beta, p-ERK2, and total ERK 1/2 proteins. In turn, DHA-high fish oil attenuated the changes induced by HS on p-4EBP1 and total ERK1 levels [7]. The results herein reported (UPR and autophagy markers) and ones previously published (muscle size, proteasome activity, and protein synthesis/degradation) contribute to the understanding of the effects of FOs supplementation on skeletal muscle atrophy due to disuse.

## 2. Materials and Methods

### 2.1. Animals

We obtained eight-week-old male Wistar rats (weighing 203 ± 20.1 g) from the Institute of Biomedical Sciences of University of São Paulo (ICB-USP). We maintained the rats under a 12-h light/dark cycle with free access to water and food. We followed the Guide for Care and Use of Laboratory Animals (National Academy of Sciences, Washington, DC, USA). The ICB-USP Ethics Committee approved the study (24/13/CEUA). The same animals and study design was used in a previous study [7].

### 2.2. Experimental Study Design

We randomly divided the rats into six groups of 10 each, totaling 60 in the whole study. The groups are: MO-C, control receiving mineral oil—MO; MO-HS, HS receiving MO; EPA-C, control treated with high EPA fish oil; EPA-HS, HS treated with high EPA fish oil; DHA-C, control treated with high DHA fish oil; and DHA-HS, HS treated with high DHA fish oil. We administered the oils (0.3 mL per 100 g weight) daily for four weeks by gavage (for details of the fatty acid composition of each FO used, please see ref. [7]). The HS was initiated at the end of the second week and continued concomitantly with the treatments with FOs for another two-week period. We removed soleus and gastrocnemius muscles from both limbs in the conditions previously described [7]. The soleus muscle displays more significant mass loss than the extensor digitorum longus in HS [7].

### 2.3. Hindlimb Suspension (HS)

The HS protocol involved attaching the rat’s tail to a rolling pulley at the top of the cage and suspending the hind limbs (30° suspension) with tape. In this situation, the animals can still move using the forelimbs but do not use their hindlimbs. The same HS protocol was used in previous studies from the same laboratory [6,7,52,53].

### 2.4. Gastrocnemius Muscle Fatty Acid Determination

We measured fatty acid composition as previously reported [7,54,55]. Gastrocnemius muscle was analyzed instead of the soleus due to the limited number of samples.

### 2.5. Mesurements of p-IRE1, JNK 1/2, p38, BiP, PDI, CHOP, and Caspase 3 Contents

We prepared soleus muscle extract [7] and measured protein content [56]. Forty µg protein was separated as previously reported [7] and results normalized by total loading of proteins [7,57] (Supplemental Figure S1). We expressed the results as relative to MO-C. We used a mixture of samples collected from all groups as a pool sample and added to all western blot runs as a normalization factor between membranes (Supplemental Figure S2). The primary antibodies included phospho-IRE 1 at S724 (p-IRE1, ab48187, Abcam, Cambridge, MA, USA), phospho-JNK 1/2 at Thr 183/Tyr 185 (p-JNK 1/2, 9251, Cell Signaling Technology—CST, Beverly, Massachusetts, USA), total JNK 1/2 (9252, CST), p-p38 at Thr 180/Tyr 182 (9216, CST), total p38 (9212, CST), BiP (3183, CST), Protein Disulfide Isomerase (PDI, 3501, CST), CHOP (2895, CST), and caspase 3 (9662, CST).

### 2.6. XBP-1 mRNA Splicing

We used TRIzol reagent (Invitrogen, Thermo Fisher Scientific, Waltham, MA, USA) and RNeasy Mini Kit (Qiagen, Hilden, Germany) to prepare total RNA from soleus (Chomczynski and Sacchi, 1987). RNA was transcribed to complementary deoxyribonucleic acid (cDNA) with the High-Capacity cDNA reverse transcription kit (Applied Biosystems, Waltham, MA, USA). XBP-1 cDNA was amplified with OneStep reverse transcription polymerase chain reaction (RT-PCR) kit (Qiagen) using primers excised by IRE1 exonuclease. Primer sequences for rat XBP-1 were 5′-AAACAGAGTAGCAGCACAGACTGC-3′ and 5′-TCCTTC TGGGTAGACCTCTGGGAG-3′. The temperatures (°C) and times (minutes) used for RT-PCR were, respectively: 50-30; 95-15; 35 cycles at 94-1, 55-1, 72-1; and 72-10. RT-PCR products were resolved, and the bands visualized, as previously described [58].

### 2.7. Real-Time Polymerase Chain Reaction (RT-PCR)

Total RNA was prepared as described above. We assessed purity through 260/280 nm absorbance ratio and RNA concentration using 260 nm absorbance. We used 1.0 μg total RNA to synthesize cDNA with the High-Capacity kit (Invitrogen). The following primers were used: GRP78 (BiP) 5’-GACGCACTTGGAATGACCCTTC-3′ and 5′-TGGCAAGAACTTGATGTCCTGC-3′; C/EBP homologous protein (CHOP) 5′-ACGGAAACAGAGTGGTCAGTGC-3′ and 5′-TGCTCCTTCTCCTTCATGCG-3′; IRE1 5′-TGTGGAGCAGAAGGACTTCGC-3′ and 5′-TCTGATGAAGCAAGGTGATGGG-3′; PERK 5′-CAAGCCAGAGGTGTTTGGGAAC-3′ and 5′-TCTCCGTCCAGGGAAGGAATG; Beclin 5′-AGCACGCCATGTATAGCAAAGA-3′ and 5′-GGAAGAGGGAAAGGACAGCAT-3′; LC3b 5′-CCAAGCCTTCTTCCTCCTGG-3′ and 5′-TCTCCTGGGAGGCATAGACC-3′; ATG14 5′-TGCCGAACAATGGGGACTAC-3′ and 5′-AGGCAGGGTTGTTATGCTCC-3′. We used SyBR Green JumpStart kit (Sigma Aldrich, Merck, St. Louis, Missouri United States) and Rotor-Gene 6000 instrument (Corbett Research, Mortlake, Australia). RPL37A (5′-CGCTAAGTACACTTGCTCCTTCTG-3′ and 5′-GCCACTGTTTTCATGCAGGAAC-3′) and HPRT1 (5′-GCGAAAGTGGAAAAGCCAAGT-3′ and 5′-GCCACATCAACAGGACTCTTTAG-3′) were used as internal controls to measure gene expression by the 2^−^^ΔΔC^_T_ method [59,60].

### 2.8. Statistical Analysis

Results are reported as the mean ± standard error of the mean (SEM). We used two-way analysis of variance (ANOVA) to indicate significant effects (*p* < 0.05) of FO treatments and HS. A Bonferroni post hoc test indicated differences between groups (GraphPad Prism software version 4.01; El Camino Real, CA, USA) when a significant interaction was found. Grubbs’ test-GraphPad software (graphpad.com/quickcalcs/Grubbs1.cfm) indicated outliers.

## 3. Results

### 3.1. Evidence for Incorporation of ω-3 FAs in Skeletal Muscle

Reductions of ω-6 FAs (MO: 28%; EPA: 14%; DHA: 7%) and ω-3 FAs (MO: 32%; EPA: 3%; DHA: 4%) observed in the HS groups were less pronounced in rats treated with FOs. In addition to the above, ω-6/ω-3 FAs ratios were lowered by the supplementation (fish oil supplementation effect, *p* < 0.001) (Supplemental Table S1 and Supplemental Figure S3).

### 3.2. IRE1 Signaling in the Soleus Muscle

**Figure 1:**
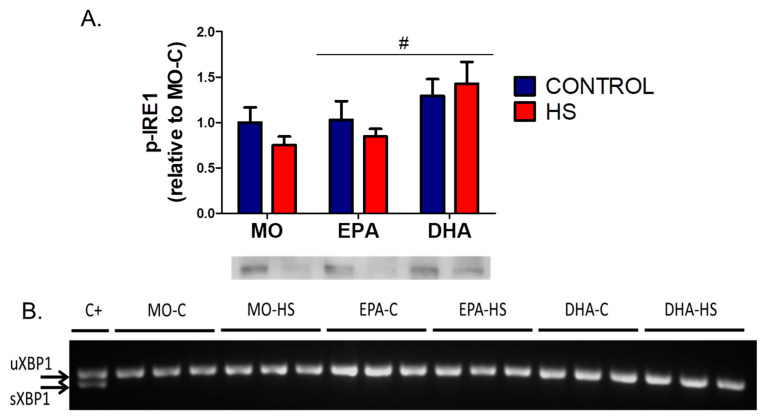
(**A**) Soleus phosphorylated IRE 1 (p-IRE1) content. Membranes stained with Ponceau S to assess total protein loading (*n* = 7–8). Results (mean ± SEM) were compared using two-way ANOVA. # For treatments with fish oils (*p* < 0.05). The six groups are: MO-C; control receiving mineral oil-MO, MO-HS; HS receiving MO, EPA-C; control treated with high EPA fish oil, EPA-HS; HS treated with high EPA fish oil, DHA-C; control treated with high DHA fish oil, and DHA-HS; HS treated with high DHA fish oil. (**B**) Complementary cDNA bands of not spliced XBP1 (uXBP1) (top band) and spliced XBP1 (sXBP1) (bottom band). Results obtained from 3 independent experiments. C+**:** positive control (insulin-producing beta-cell line, INS-1E treated with 1 μM thapsigargin, 1 h). MO: Mineral oil supplementation; EPA: High eicosapentaenoic acid fish oil supplementation; DHA: High docosahexaenoic acid fish oil supplementation; HS: hindlimb suspension; SEM: Standard Error of the Mean.

Using western blot analysis and RT-PCR, p-IRE1 protein content (Supplemental Figure S4) and sXBP1 mRNA levels were measured, respectively. As described above, IRE1α cleaves XBP1 mRNA removing 26 nucleotides and generating the spliced XBP1 (sXBP1) that promotes transcription of UPR genes. Treatment with FOs showed higher values in p-IRE1 protein content (Figure 1A). sXBP1 was detected in the positive control but it was not found in any experimental group (Figure 1B). 

**Figure 2:**
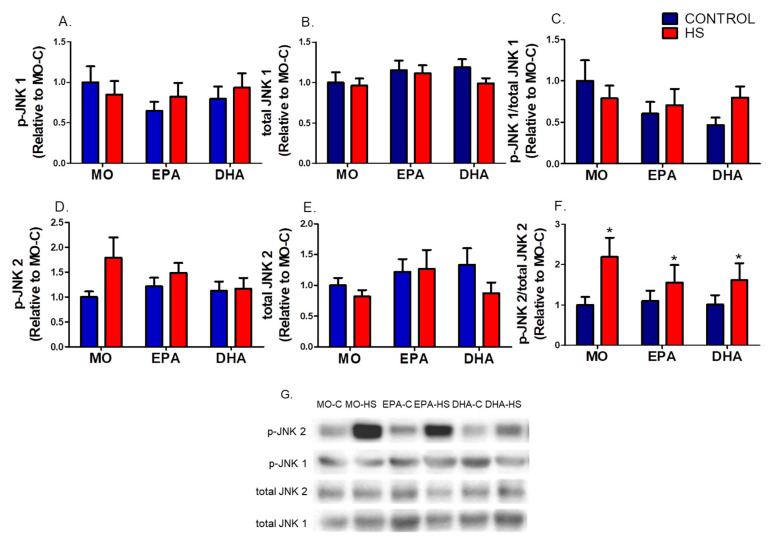
Soleus phosphorylated and total JNK 1 and 2 contents: (**A**) p-JNK 1, (**B**) total JNK 1, (**C**) p-JNK 1/total JNK 1 ratio, (**D**) p-JNK 2, (**E**) total JNK 2, (**F**) p-JNK 2/total JNK 2 ratio, (**G**) western blotting representative images of JNK 1 and 2. Results (mean ± SEM) were compared using two-way ANOVA (*n* = 7–8). * Hindlimb suspension effect (*p* < 0.05). The six groups are: MO-C; control receiving mineral oil-MO, MO-HS; HS receiving MO, EPA-C; control treated with high EPA fish oil, EPA-HS; HS treated with high EPA fish oil, DHA-C; control treated with high DHA fish oil, and DHA-HS; HS treated with high DHA fish oil. MO: Mineral oil supplementation; EPA: High eicosapentaenoic acid fish oil supplementation; DHA: High docosahexaenoic acid fish oil supplementation; HS: hindlimb suspension; SEM: Standard Error of the Mean.

The contents p-JNK 1, total JNK1, p-JNK2, and total JNK2 contents, and p-JNK 1/total JNK1 ratio were not significantly different among the groups (please see Figure 2 and Supplemental Figure S4). However, p-JNK2/total JNK2 ratio was significantly higher (*p* ˂ 0.05) in MO-HS (119%), EPA-HS (42%), and DHA-HS (59%) than in non-HS rats (hindlimb suspension effect, Figure 2F).

**Figure 3:**
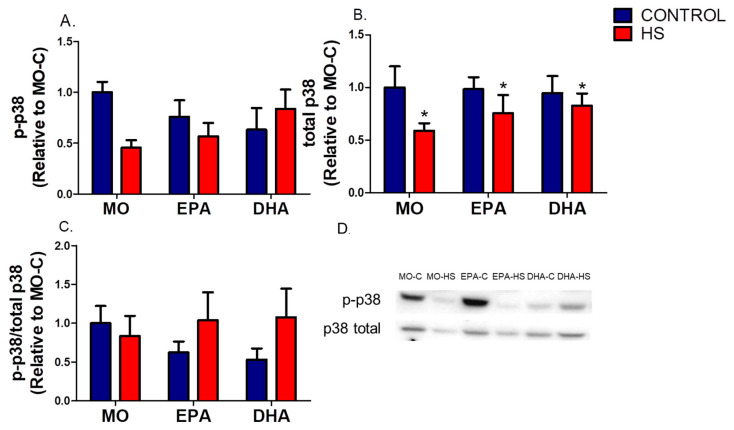
Soleus p38 protein content. (**A**) Phosphorylated p-38 (p-p38), (**B**) total p38, (**C**) p-p38/total p38 ratio, (**D**) western blotting representative images of p38. Results (mean ± SEM) were compared using two-way ANOVA (*n* = 6–8). * Hindlimb suspension effect (*p* < 0.05). The six groups are: MO-C; control receiving mineral oil-MO, MO-HS; HS receiving MO, EPA-C; control treated with high EPA fish oil, EPA-HS; HS treated with high EPA fish oil, DHA-C; control treated with high DHA fish oil, and DHA-HS; HS treated with high DHA fish oil. MO: Mineral oil supplementation; EPA: High eicosapentaenoic acid fish oil supplementation; DHA: High docosahexaenoic acid fish oil supplementation; HS: hindlimb suspension; SEM: Standard Error of the Mean.

The p-p38 content and the p-p38/total p38 ratio were not different (*p* > 0.05, please see Figure 3 and Supplemental Figure S4). However, total p38 content was significantly lower (hindlimb suspension effect, *p* ˂ 0.05) in the MO-HS (41%), EPA-HS (23%), and DHA-HS (13%) than in the non-HS groups (Figure 3B).

### 3.3. Soleus ERS Markers

**Figure 4:**
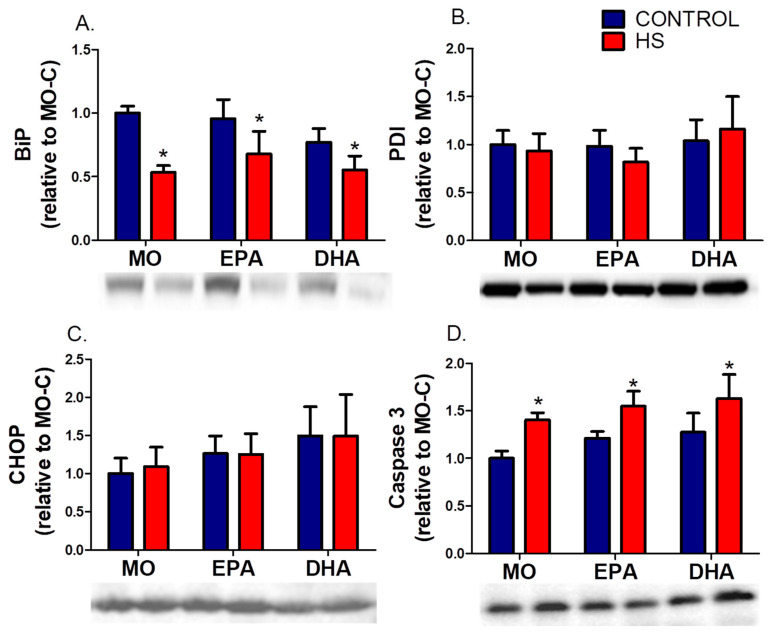
Soleus content of endoplasmic reticulum stress markers: (**A**) binding immunoglobulin protein, BIP, (**B**) protein disulfide isomerase, PDI, (**C**) CCAAT/enhancer-binding protein homologous protein, CHOP, (**D**) caspase 3 (intact protein form). Results (mean ± SEM) were compared using two-way ANOVA (*n* = 6–8). * Hindlimb suspension effect (*p* < 0.05). The six groups are: MO-C; control receiving mineral oil-MO, MO-HS; HS receiving MO, EPA-C; control treated with high EPA fish oil, EPA-HS; HS treated with high EPA fish oil, DHA-C; control treated with high DHA fish oil, and DHA-HS; HS treated with high DHA fish oil. MO: Mineral oil supplementation; EPA: High eicosapentaenoic acid fish oil supplementation; DHA: High docosahexaenoic acid fish oil supplementation; HS: hindlimb suspension; SEM: Standard Error of the Mean.

HS reduced (*p* ˂ 0.01) soleus BiP protein content (Figure 4) with greater change in the MO group (~−47%). On the other hand, PDI and CHOP protein content remained unchanged in all groups (*p* > 0.05, Figure 4B,C). Caspase 3 content was higher (hindlimb suspension effect, *p* ˂ 0.01) in the MO-HS (40%), EPA-HS (28%) and DHA-HS (28%) than in non-HS animals (Figure 4D and Supplemental Figure S4).

### 3.4. Soleus mRNA Expression (RT-PCR) of ER Stress and Autophagy Markers

**Figure 5:**
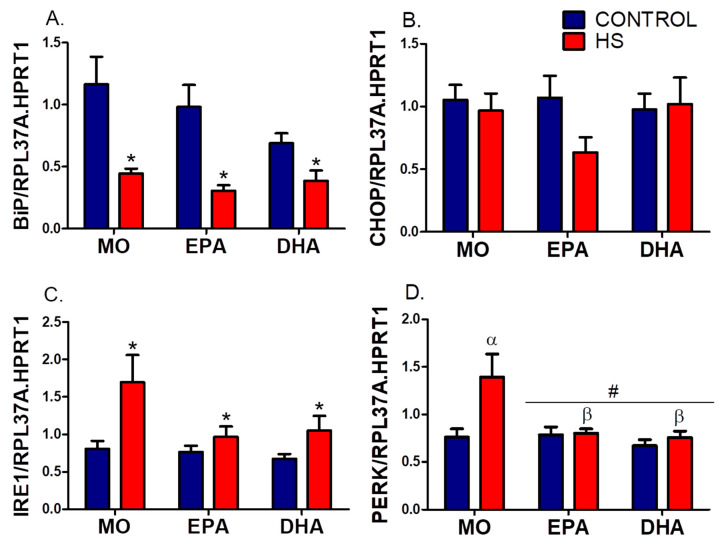
Effects of hindlimb suspension (HS) and oral fish oil (FO) supplementations (High EPA or High DHA) on soleus expression of endoplasmic reticulum stress markers: (**A**) BiP, (**B**) CHOP, (**C**) IRE1, (**D**) PERK. Results are reported as mean ± SEM (*n* = 6–9). RPL37A and HPRT1 genes are internal controls. BiP, CHOP, and IRE1 results were analyzed using two-way ANOVA. * HS effect (*p* < 0.05). PERK results were analyzed using two-way ANOVA and Bonferroni post hoc test. # FO treatment effects (*p* < 0.05). α Different from MO-C (*p* < 0.001). β Different from MO-HS (*p* < 0.01). The six groups are: MO-C, control receiving mineral oil-MO; MO-HS, HS receiving MO; EPA-C, control treated with high EPA fish oil; EPA-HS, HS treated with high EPA fish oil; DHA-C, control treated with high DHA fish oil; and DHA-HS, HS treated with high DHA fish oil. MO: Mineral oil supplementation; EPA: High eicosapentaenoic acid fish oil supplementation; DHA: High docosahexaenoic acid fish oil supplementation; HS: hindlimb suspension; SEM: Standard Error of the Mean.

**Figure 6:**
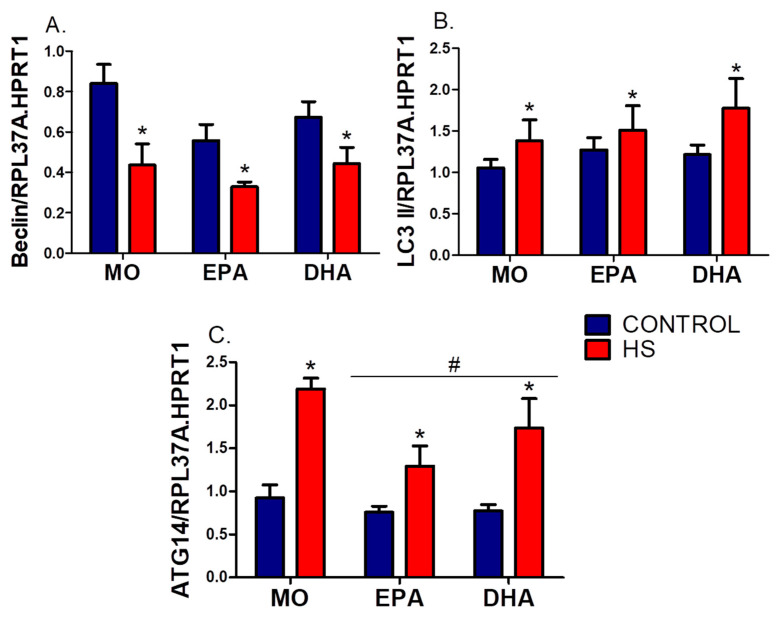
Effects of hindlimb suspension (HS) and oral fish oil (FO) supplementations (High EPA or High DHA) on autophagy gene expressions: (**A**) Beclin, (**B**) LC3 II, (**C**) ATG14. Results (mean ± SEM) were compared using two-way ANOVA (*n* = 6–9). * HS effect (*p* < 0.05). # FO treatment effect (*p* < 0.05). The six groups are: MO-C; control receiving mineral oil-MO, MO-HS; HS receiving MO, EPA-C; control treated with high EPA fish oil, EPA-HS; HS treated with high EPA fish oil, DHA-C; control treated with high DHA fish oil, and DHA-HS; HS treated with high DHA fish oil. MO: Mineral oil supplementation; EPA: High eicosapentaenoic acid fish oil supplementation; DHA: High docosahexaenoic acid fish oil supplementation; HS: hindlimb suspension; SEM: Standard Error of the Mean.

Expressions of UPR markers (BiP, CHOP, IRE1, and PERK) are in Figure 5, and Supplemental Figure S5 and of autophagy markers (Beclin, LC3b, and ATG14) are in Figure 6 and Supplemental Figure S5). HS decreased (*p* ˂ 0.001) BiP expression, with the DHA-C being 42% lower than the MO-C group (MO-C, 1.2 ± 0.2 vs. DHA-C 0.7 ± 0.1, *p* ˂ 0.05, Figure 5A). CHOP expression remained unchanged (*p* > 0.05, Figure 5B). In contrast, IRE1 expression was higher in HS (*p* ˂ 0.01), being more prominent in the MO group (MO: 111%, EPA: 27%, and DHA: 56%), than in non-HS rats (Figure 5C). PERK expression was changed by FO treatments (*p* ˂ 0.01) and HS (*p* ˂ 0.05), with significant differences between groups (interaction effect, *p* ˂ 0.05). PERK expression was augmented in MO rats (MO-C vs. MO-HS, *p* ˂ 0.001), an effect abolished by both FOs; MO-HS: 1.4 ± 0.2; EPA-HS: 0.8 ± 0.0; DHA-HS: 0.7 ± 0.1, MO-HS vs. EPA-HS, *p* ˂ 0.01 and MO-HS vs. DHA-HS, *p* ˂ 0.001 (Figure 5D).

Beclin expression decreased significantly by HS in all groups (hindlimb suspension effect, *p* ˂ 0.001), with a prominent level in MO-C (MO-C: 0.8 ± 0.1, EPA-C: 0.6 ± 0.1, and DHA-C: 0.7 ± 0.1) (Figure 6A). Two-way ANOVA detected significant effect of HS on LC3b expression (hindlimb suspension effect, *p* ˂ 0.05), which was increased in the MO-HS (31%), EPA-HS (19%) and DHA-HS (46%) groups when compared to the non-HS animals (Figure 6B). Additionally, ATG14 expression was significantly increased by the HS (hindlimb suspension effect, *p* ˂ 0.05) with attenuation in this effect by the FO supplementations (fish oil supplementation effect, *p* ˂ 0.001) (Figure 6C).

## 4. Discussion

It has been reported that HS mimics spaceflight, bed rest, or the hospitalization state in humans [52]. Previous studies of our group [6,7,53] and others [61,62] described that this experimental model promotes a reduction of body weight, fat mass, muscle weight, and soleus CSA. Concomitantly, there are a drop of protein synthesis and a raise of protein degradation. Previously, we showed that in rats subjected to HS, FOs increase CSA of soleus muscle fibers. Separately, high EPA FO supplementation attenuates the increase in 26S proteasome activity and the decrease on protein synthesis markers. Conversely, high DHA FO supplementation has fewer molecular effects in the protein synthesis pathway [7].

In the skeletal muscle, the endoplasmic reticulum regulates calcium concentrations during muscle contractions and plays a critical role in cellular homeostasis [63]. However, few studies have investigated strategies to prevent alterations on ERS and autophagy markers in a muscle disuse condition. The effects of ω-3 FAs (EPA and/or DHA) attenuate ERS and autophagy markers expressions induced by HS were investigated in the present study. HS altered the soleus protein content (decreasing total p38 and BiP and increasing p-JNK2/total JNK2 ratio and caspase 3) and gene expressions (decreasing BiP and increasing IRE1 and PERK) of ERS and autophagy (decreasing Beclin and increasing LC3 and ATG14) markers. The treatment with both FOs decreased the ω-6/ω-3 FAs ratio in the skeletal muscle. In addition, both FOs attenuated the decrease in p-IRE1 content and the increase in PERK and ATG14 mRNA expressions induced by HS, the last two displaying particularly meaningful changes as compared to the other measurements. Although in this study the effect of the groups as a whole must be considered, we also performed an unpaired *t*-test to examine the effects of HS in each condition (MO, EPA or DHA). We observed that fish oils can attenuate the protein contents changes on p-p38, BiP and caspase 3, and gene expressions of IRE1, PERK, and Beclin induced by two-week HS.

Herein, there was no evidence of XBP1 splicing in any group. However, there was a percentage increase in p-IRE1 protein content in the DHA-HS compared to the DHA-C group. It is known that the IRE1 pathways can also activate apoptosis through JNK activation [24]. In this sense, it is important to point out that an increase in p-JNK 2/total JNK 2 ratio in HS rats was found. Another MAPK family member is the p38, which is activated by inflammatory signals and oxidative stress, and it is involved in muscle atrophy [64,65]. p38 activates transcriptional factors that lead to protein degradation and apoptosis in skeletal muscle [66]. While it was not observed increased p38 activation, a decrease in total p38 content was detected in the HS groups. This reduction in p38 content could account for the observed increase in JNK activity. Notably, an increase in caspase 3 content was detected in all HS groups after 14 days, indicating apoptotic pathway activation.

It is well-known that BiP is an essential protein in the UPR and ERS. The release of BiP from the luminal domain of IRE1, PERK, or ATF6 correlates with an imbalance in the ER microenvironment and activation of the three signaling pathways. High BiP levels could delay the UPR, and low levels could prolong it [67]. Woodworth-Hobbs et al. (2017) reported that muscle atrophy induced by palmitate can be prevented by DHA through an ERS/UPR mechanism. DHA was postulated to activate proteolysis via caspase and to augment expressions of autophagy-associated genes [68]. Herein, decreased BiP expression and protein content and increased IRE1 and PERK expression in the soleus of MO-HS animals were observed, which is indicative of a prolonged ERS. Both FOs suppressed the upregulation of IRE1 and PERK expression. This finding may have a significant effect at the beginning of the UPR signaling, but since there were no differences between groups regarding JNK activation and caspase 3, PDI, and CHOP expression or content, this effect was not translated into significant downstream alterations. 

It was previously reported by Chen et al. (2015) that transgenic amyotrophic lateral sclerosis (ALS) mice (G93A*SOD1 heterozygote) displayed increased levels of ERS markers in the white gastrocnemius fibers. These authors found more pronounced effects than those reported in the present study, which is probably due to the ALS-related neurological changes (e.g., degenerative neuromuscular disease) [69]. In addition, the white muscle portion has more glycolytic/type II fibers with less oxidative capacity, whereas the soleus, herein studied, has predominantly oxidative/type I red fibers [70]. Differences between skeletal muscles with different fiber type compositions require further investigation. 

Baehr et al. [42] investigated the effects of HS for 14 days followed by the same period of reloading on 9 (adult) and 29 (aged) months old male F344BN rats. ERS markers (BiP, PDI, CHOP) contents did not change by HS [42]. These results are consistent with PDI and CHOP content results herein observed but are in contrast with the decrease in BiP protein content and gene expression described. Similarly, the authors observed no changes in autophagy markers (phospho-Unc-51 like autophagy activating kinase-1 [p-ULK1], p62, ATG7, Beclin, and LC3b-II) in soleus. Despite showing no evidence of autophagy or ERS increase in the soleus of 9-month-old animals during HS, their data suggest that these processes are activated upon reloading [42], contrary to what was herein found. These differences could be due to the strain of rat employed (F344BN vs. Wistar) or animal age (9 months vs. 2 months). 

Herein, Beclin, LC3b, and ATG14 expressions, markers of the macroautophagy, were evaluated [33]. All HS groups exhibited increased ATG14 and LC3b expressions in the soleus muscle, markers commonly upregulated during autophagy. However, contrary to the hypothesis of the present study, the expression of Beclin was reduced by the HS. In the dynamics of autophagosome formation, ATG14 recruits PI3K-III complex when subjected to autophagic stress [71,72,73,74]. In addition, it is known that the Beclin homodimer is inactive when bound to the BCL2-complex. However, when BCL2 is phosphorylated, Beclin is released, allowing it to bind to ATG14, VPS34 and VPS15 [71]. Therefore, a concomitant increase in ATG14 and Beclin expression was expected to be observed. However, this was not the case.

Considering solely the gene expression, without protein translation or post-translational modifications, stability changes, protein translocation and assembly of the autophagy complex, there was another study reporting a disconnection between the expression of Beclin and ATG14. In HeLa cells, siRNA-mediated knockdown of either Beclin or ATG14 did not affect the mRNA levels of the other gene [75]. Moreover, there are different transcription factors regulating Beclin1 and ATG14. While forkhead box protein [FoxO] can affect both, others such as nuclear factor-kappa B [NF-κB], farnesoid X Receptor [FXR], peroxisome proliferator-activated receptor alpha [PPARα], N-terminal p63 isoform alpha [ΔNp63α] and activator of transcription 1 [STAT-1] were described for Beclin only (reviewed by Füllgrabe et al.) [76]. Thus, it is possible that an extreme condition like hindlimb suspension, which modulates several signaling pathways, would interfere with the activity/expression of some of those transcription factors, with independent effects in the expression of Beclin and ATG14. In this work, a part of the entire process was evaluated, and other markers can influence this pathway. The increase in ATG14 and LC3 could have occurred through the PERK-eIF2α-ATF4 pathway [23]. As mentioned above, ERS and autophagy are involved in controlling skeletal muscle mass loss. Despite some markers of these pathways being upregulated, their specific activation during muscle unloading requires further investigation. For example, the involvement of 5′ adenosine monophosphate-activated protein kinase [AMPK], a serine/threonine protein kinase/mammalian target of rapamycin complex [Akt/mTORC], FoxO3, NF-κB, and protein kinase C theta [PKCθ] should be considered [77,78,79,80].

## 5. Strengths and Limitations

The anabolic effects of ω-3 FAs on protein synthesis were previously described [13]. There is evidence that FO supplementation can improve insulin sensitivity and increase the protein synthesis pathway activity. FO can also attenuate some markers of the UPR in ERS-induced conditions (ex. PERK), which is in contrast to the induction of ER ceramide synthesis by palmitic acid [81]. Herein, high EPA and high DHA FOs attenuated the increased expression of PERK induced by HS. Such an effect could potentially prevent the deleterious activation of UPR and ERS during disuse muscle-induced atrophy.

It is important to point out that our study has some limitations. For example, the experimental protocol was conducted over a fixed period, with supplementation for four weeks and HS in the final two weeks. The authors interpreted that the effects of the FOs were mainly due to an increase in the content of ω-3 FAs, but one cannot rule out a decrease in the proportion of saturated fatty acids plays a role in the results reported. Additionally, we did not perform any of the analyses on skeletal muscles with different fiber type compositions. Some autophagy and ERS markers were not evaluated, and some were evaluated through their gene expression and not their protein content. A possible total JNK reduction in the DHA group may account for the increased phospho/total ratio Further research is necessary to detail how FOs could potentially attenuate ERS and autophagy activities in muscles undergoing atrophy. The supplementation in different doses of FO in patients under a condition of muscle disuse should be evaluated and muscle ERS and autophagy markers should be measured. Additionally, studies measuring protein synthesis/degradation in vivo will be of great interest. However, despite the mentioned limitations, the results are consistent and support the conclusions of the study.

## 6. Conclusions

Both FOs attenuated the increase in PERK and ATG14 expressions induced by HS. Thus, both FOs could potentially attenuate ERS and autophagy in skeletal muscles undergoing atrophy.

## Supplementary Materials

The following are available online at https://www.mdpi.com/article/10.3390/nu13072298/s1, Figure S1: Ponceau S quantification of western blot membranes, Figure S2: Images used for quantitative analysis of the western blot assays in this study, Figure S3: Results of the analysis of fatty acids composition in the gastrocnemius muscle used in the Supplemental Table S1 of this study, Figure S4: Results of the quantitative analysis of western blot assays used in the Figure 1, Figure 2, Figure 3 and Figure 4 of this study, Figure S5: RT-PCR results after 2^−^^ΔΔC^_T_ calculation for the relative expression of genes used in the Figure 5; Figure 6 of this study, Table S1: Composition of fatty acids in g/100 g gastrocnemius muscle wet weight.
