# Supplementary material for: Endoplasmic Reticulum Stress and Autophagy Markers in Soleus Muscle Disuse-Induced Atrophy of Rats Treated with Fish Oil
Source: Nutrients. 2021 Jul 3;13(7):2298. doi: 10.3390/nu13072298 (PMC8308346; doi:10.3390/nu13072298)
Supplement: Supplementary file 1 [file nutrients-13-02298-s001.zip › nutrients-1257594-supplementary.pdf]

Supplemental Table S1. Composition of fatty acids in g/100 g gastrocnemius muscle wet weight.

Fatty acid	Name	MO-C	MO-HS	EPA-C	EPA-HS	DHA-C	DHA-HS
**14 : 0**	Myristic	—	—	—	—	—	—
**16 : 0**	Palmitic	0.241 ± 0.067	0.182 ± 0.041	0.187 ± 0.040	0.160 ± 0.008	0.179 ± 0.020	0.176 ± 0.028
**16 : 1 (n-7)**	Hexadecenoic	—	0.009 ± 0.006	0.007 ± 0.006	—	0.006 ± 0.005	0.008 ± 0.007
**17 : 0**	Margaric	—	—	0.004 ± 0.004	—	—	—
**17 : 1 (n-7)**	Heptadecenoic	—	—	—	—	—	—
**18 : 0**	Stearic[#]	0.158 ± 0.028	0.112 ± 0.008	0.114 ± 0.018	0.112 ± 0.011	0.105 ± 0.019	0.102 ± 0.009
**18 : 1 (n-9)**	Oleic	0.153 ± 0.082	0.108 ± 0.054	0.112 ± 0.027	0.080 ± 0.021	0.099 ± 0.014	0.097 ± 0.016
**18 : 1 (n-7)**	Vaccenic[#]	0.032 ± 0.007	0.024 ± 0.007	0.021 ± 0.005	0.020 ± 0.002	0.022 ± 0.001	0.021 ± 0.003
**18 : 2 (n-6)**	Linoleic	0.332 ± 0.142	0.220 ± 0.086	0.208 ± 0.067	0.160 ± 0.009	0.201 ± 0.039	0.183 ± 0.026
**20 : 0**	Eicosanoic	—	—	—	—	—	—
**20 : 1 (n-9)**	Eicosenoic	—	—	—	—	—	—
**18 : 3 (n-6)**	γ -Linolenic	—	—	—	—	0.001 ± 0.000	—
**18 : 3 (n-3)**	α-Linolenic*	0.040 ± 0.034	0.009 ± 0.005	0.006 ± 0.006	—	0.015 ± 0.012	0.005 ± 0.004
**22 : 0**	Docosanoic	0.025 ± 0.0024	—	—	—	—	—
**20 : 2**	Eicosadienoic	—	—	—	0.002 ± 0.002	—	—
**20 : 3 (n-6)**	Eicosatrienoic	—	—	—	—	—	—
**20 : 4 (n-6)**	Arachidonic[###]	0.165 ± 0.015	0.136 ± 0.003	0.079 ± 0.009	0.081 ± 0.011	0.076 ± 0.008	0.074 ± 0.007
**20 : 5 (n-3)**	Eicosapentaenoic	—	—	—	0.022 ± 0.003	0.007 ± 0.001	0.007 ± 0.002
**24 : 1 (n-9)**	Nervonic	0.018 ± 0.013	0.002 ± 0.002	—	0.006 ± 0.002	0.007 ± 0.007	0.003 ± 0.003
**22 : 5 (n-6)**	Docosapentaenoic	0.014 ± 0.003	0.013 ± 0.001	—	0.003 ± 0.003	0.006 ± 0.001	0.006 ± 0.001
**22 : 5 (n-3)**	Docosapentaenoic[###]	0.021 ± 0.002	0.017 ± 0.002	0.034 ± 0.006	0.031 ± 0.002	0.011 ± 0.002	0.010 ± 0.002
**22 : 6 (n-3)**	Docosahexaenoic[###]	0.071 ± 0.010	0.064 ± 0.003	0.126 ± 0.018	0.138 ± 0.027	0.173 ± 0.034	0.176 ± 0.026
**Total**	Saturated	0.424 ± 0.111	0.295 ± 0.046	0.305 ± 0056	0.277 ± 0.017	0.284 ± 0.034	0.279 ± 0.035

	Monounsaturated	0.203 ± 0.099	0.143 ± 0.065	0.140 ± 0.037	0.107 ± 0.025	0.133 ± 0.011	0.129 ± 0.028
	Polyunsaturated	0.642 ± 0.188	0.459 ± 0.097	0.483 ± 0.113	0.437 ± 0.043	0.488 ± 0.081	0.461 ± 0.066
	Omega-6[##]	0.511 ± 0.152	0.369 ± 0.088	0.287 ± 0.076^{x}	0.247 ± 0.013	0.283 ± 0.044^{x}	0.263 ± 0.034
	Omega-3[###]	0.132 ± 0.038	0.090 ± 0.009	0.196 ± 0.037	0.191 ± 0.030^{y}	0.206 ± 0.042^{x}	0.198 ± 0.033^{y}
**Fatty %**		1.360 ± 0.419	0.963 ± 0.223	0.991 ± 0.220	0.878 ± 0.047	0.969 ± 0.128	0.929 ± 0.131

The determination of fat was calculated from the tridecanoate triglyceride, which was used as internal standard. Values are presented as mean ± SD, n=3 animals. The results were compared using two-way ANOVA. The results were compared using two-way ANOVA. * *P*<0.05: hindlimb suspension main effect. # *P*<0.05; ## *P*<0.01; ### *P*<0.001: fish oil supplementation main effect. **MO-C:** Mineral oil supplemented group; **MO-HS:** Mineral oil supplemented and hindlimb suspension group; **EPA-C:** High eicosapentaenoic acid fish oil supplemented group; **EPA-HS:** High eicosapentaenoic acid fish oil supplemented and hindlimb suspension group; **DHA-C:** High docosahexaenoic acid fish oil supplemented group; **DHA-HS:** High docosahexaenoic acid fish oil supplemented and hindlimb suspension group; — : not detected.

Supplemental Figure S1

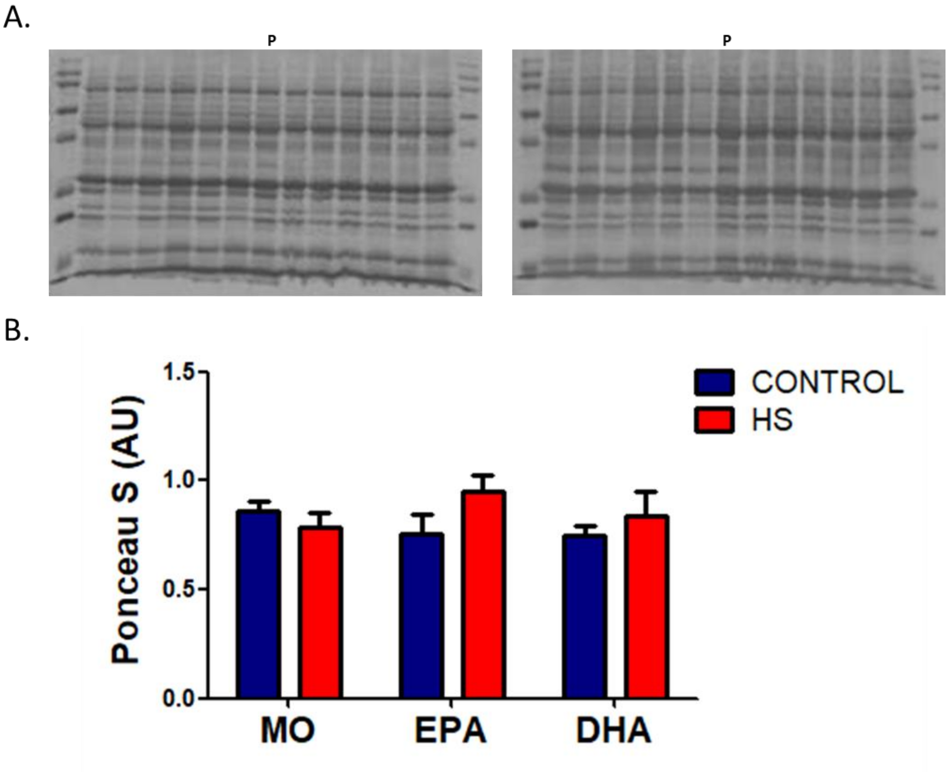

Supplemental Figure S1. Ponceau S quantification of western blot membranes. (A) Representative images of the western blot membranes stained with Ponceau S used in this study. (B) Average quantitative analysis of Ponceau S staining. No significant differences were observed. Results were compared using two-way ANOVA (hindlimb suspension effect) and the Bonferroni post-hoc test. The six groups included mineral oil supplemented control (MO-C), MO with hind limb suspension (MO-HS), high EPA fish oil control (EPA-C), EPA with HS (EPA-HS), high DHA fish oil control (DHA-C) and DHA with HS (DHA-HS). P, pool containing a mixture with equal parts of all samples – used to normalize Ponceau S quantitative results; AU, arbitrary units.

Supplemental Figure S2

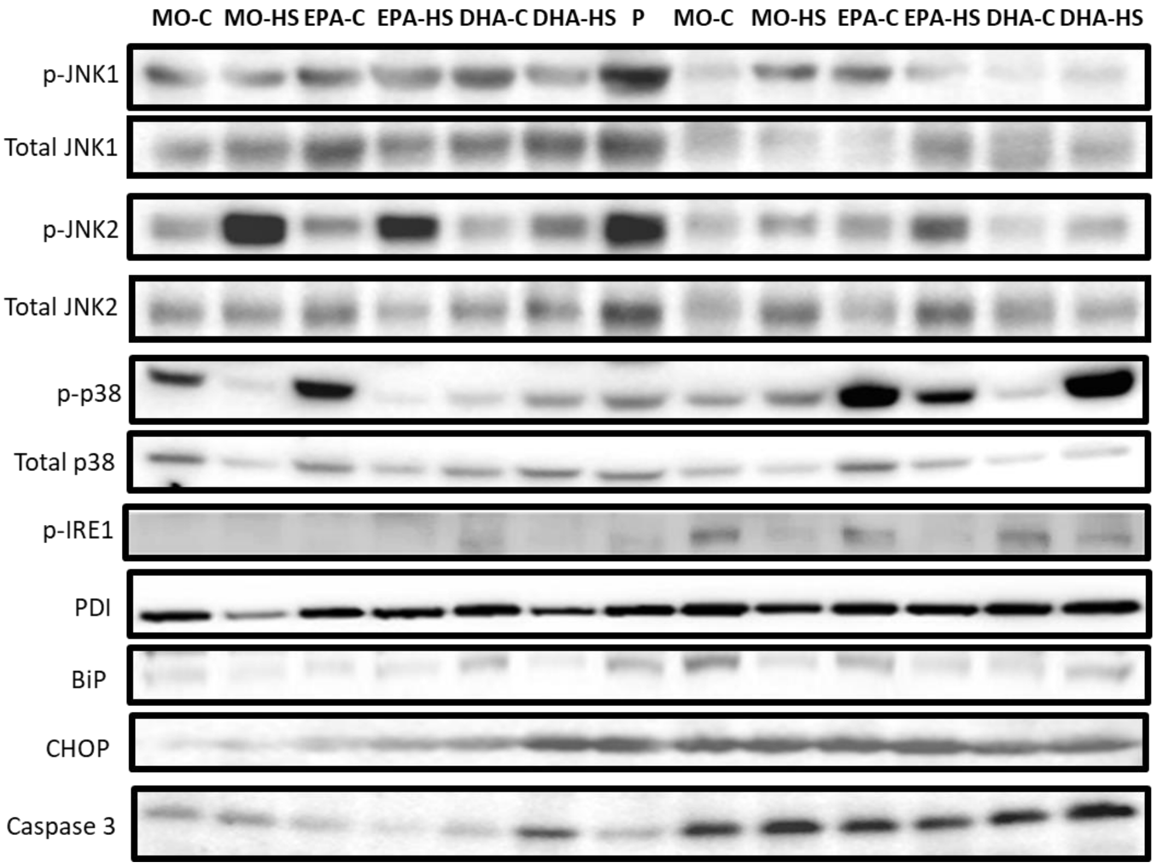

Supplemental Figure S2. Images used for quantitative analysis of the western blot assays in this study. **MO-C:** Mineral oil supplemented group; **MO-HS:** Mineral oil supplemented and hindlimb suspension group; **EPA-C:** High eicosapentaenoic acid fish oil supplemented group; **EPA-HS:** High eicosapentaenoic acid fish oil supplemented and hindlimb suspension group; **DHA-C:** High docosahexaenoic acid fish oil supplemented group; **DHA-HS:** High docosahexaenoic acid fish oil supplemented and hindlimb suspension group; **P**: pool containing a mixture of equal parts of all samples – used to normalize Ponceau S quantitative results.

Supplemental Figure S3

	MO-C			MO-HS			EPA-C			EPA-HS			DHA-C			DHA-HS		
**S**	**Pag/100g1**	**Pag/100g2**	**Pag/100g3**	**Pag/100g1**	**Pag/100g2**	**Pag/100g3**	**Pag/100g1**	**Pag/100g2**	**Pag/100g3**	**Pag/100g1**	**Pag/100g2**	**Pag/100g3**	**Pag/100g1**	**Pag/100g2**	**Pag/100g3**	**Pag/100g1**	**Pag/100g2**	**Pag/100g3**
10:00	0.000	0.000	0.000	0.000	0.000	0.000	0.000	0.000	0.000	0.000	0.000	0.000	0.000	0.000	0.000	0.000	0.000	0.000
12:00	0.000	0.000	0.000	0.000	0.000	0.000	0.000	0.000	0.000	0.000	0.000	0.000	0.000	0.000	0.000	0.000	0.000	0.000
14:00	0.000	0.000	0.000	0.000	0.000	0.000	0.000	0.000	0.000	0.000	0.000	0.000	0.000	0.000	0.000	0.000	0.000	0.000
15:00	0.000	0.000	0.000	0.000	0.000	0.000	0.000	0.000	0.000	0.000	0.000	0.000	0.000	0.000	0.000	0.000	0.000	0.000
16:00	0.303	0.248	0.171	0.159	0.158	0.229	0.145	0.192	0.225	0.169	0.155	0.157	0.201	0.163	0.172	0.204	0.176	0.148
17:00	0.000	0.000	0.000	0.000	0.000	0.000	0.004	0.009	0.000	0.000	0.000	0.000	0.000	0.000	0.000	0.000	0.000	0.000
18:00	0.186	0.157	0.131	0.104	0.113	0.119	0.097	0.112	0.132	0.124	0.102	0.109	0.112	0.084	0.120	0.112	0.094	0.100
20:00	0.000	0.000	0.000	0.000	0.000	0.000	0.000	0.000	0.000	0.000	0.000	0.000	0.000	0.000	0.000	0.000	0.000	0.000
21:00	0.000	0.000	0.000	0.000	0.000	0.000	0.000	0.000	0.000	0.000	0.000	0.000	0.000	0.000	0.000	0.000	0.000	0.000
22:00	0.028	0.047	0.000	0.000	0.000	0.000	0.000	0.000	0.000	0.000	0.000	0.000	0.000	0.000	0.000	0.000	0.000	0.000
23:00	0.000	0.000	0.000	0.000	0.000	0.000	0.000	0.000	0.000	0.000	0.000	0.000	0.000	0.000	0.000	0.000	0.000	0.000
24	0.000	0.000	0.000	0.002	0.004	0.000	0.000	0.000	0.000	0.004	0.008	0.004	0.000	0.000	0.000	0.000	0.000	0.000
	**0.517**	**0.453**	**0.302**	**0.264**	**0.274**	**0.348**	**0.246**	**0.313**	**0.357**	**0.297**	**0.265**	**0.269**	**0.314**	**0.247**	**0.292**	**0.317**	**0.270**	**0.248**
**M**																		
14:01	0.000	0.000	0.000	0.000	0.000	0.000	0.000	0.000	0.000	0.000	0.000	0.000	0.000	0.000	0.000	0.000	0.000	0.000
15:01	0.000	0.000	0.000	0.000	0.000	0.000	0.000	0.000	0.000	0.000	0.000	0.000	0.000	0.000	0.000	0.000	0.000	0.000
16:01	0.000	0.000	0.000	0.005	0.006	0.016	0.000	0.009	0.011	0.000	0.000	0.000	0.008	0.009	0.000	0.012	0.013	0.000
17:01	0.000	0.000	0.000	0.000	0.000	0.000	0.000	0.000	0.000	0.000	0.000	0.000	0.000	0.000	0.000	0.000	0.000	0.000
18:1 9c	0.224	0.173	0.063	0.078	0.077	0.171	0.082	0.121	0.133	0.065	0.104	0.071	0.114	0.096	0.087	0.104	0.108	0.079
18:1 11c	0.040	0.031	0.026	0.020	0.020	0.031	0.016	0.022	0.025	0.019	0.022	0.020	0.024	0.021	0.022	0.023	0.021	0.018
20:01	0.000	0.000	0.000	0.000	0.000	0.000	0.000	0.000	0.000	0.000	0.000	0.000	0.000	0.000	0.000	0.000	0.000	0.000
22:01	0.000	0.000	0.000	0.000	0.000	0.000	0.000	0.000	0.000	0.000	0.000	0.000	0.000	0.000	0.000	0.000	0.000	0.000
24:00	0.020	0.029	0.004	0.003	0.004	0.000	0.000	0.000	0.000	0.004	0.009	0.006	0.000	0.005	0.014	0.004	0.005	0.000
	**0.284**	**0.234**	**0.092**	**0.106**	**0.107**	**0.218**	**0.098**	**0.152**	**0.169**	**0.088**	**0.135**	**0.097**	**0.145**	**0.131**	**0.123**	**0.143**	**0.147**	**0.097**
**P**																		
18:2 c	0.477	0.326	0.194	0.178	0.163	0.319	0.137	0.216	0.271	0.162	0.168	0.151	0.246	0.172	0.184	0.206	0.188	0.154
18:3 g	0.000	0.000	0.000	0.000	0.000	0.000	0.000	0.000	0.000	0.000	0.000	0.000	0.000	0.000	0.000	0.000	0.000	0.000
**18:03 n-3**	0.060	0.059	0.000	0.007	0.005	0.015	0.000	0.008	0.011	0.000	0.000	0.000	0.009	0.007	0.029	0.008	0.007	0.000
18:04	0.000	0.000	0.000	0.000	0.000	0.000	0.000	0.000	0.000	0.000	0.000	0.000	0.000	0.000	0.000	0.000	0.000	0.000
20:2 n-7	0.000	0.000	0.000	0.000	0.000	0.000	0.000	0.000	0.000	0.002	0.003	0.000	0.000	0.000	0.000	0.000	0.000	0.000
20:3 n-6	0.000	0.000	0.000	0.000	0.000	0.000	0.000	0.000	0.000	0.000	0.000	0.000	0.000	0.000	0.000	0.000	0.000	0.000
20:4 n-6	0.179	0.149	0.166	0.133	0.137	0.139	0.071	0.077	0.089	0.092	0.070	0.082	0.077	0.066	0.083	0.082	0.072	0.068
**20:4 n-3**	0.000	0.000	0.000	0.000	0.000	0.000	0.000	0.000	0.000	0.000	0.000	0.000	0.000	0.000	0.000	0.000	0.000	0.000
**22:02**	0.000	0.000	0.000	0.000	0.000	0.000	0.000	0.000	0.000	0.000	0.000	0.000	0.000	0.000	0.000	0.000	0.000	0.000
**20:05 n-3**	0.000	0.000	0.000	0.000	0.000	0.000	0.023	0.025	0.040	0.025	0.021	0.020	0.008	0.006	0.008	0.008	0.007	0.004
**22:04**	0.000	0.000	0.000	0.000	0.000	0.000	0.000	0.000	0.000	0.000	0.000	0.000	0.000	0.000	0.000	0.000	0.000	0.000
**21:05 n-3**	0.000	0.000	0.000	0.000	0.000	0.000	0.000	0.000	0.000	0.000	0.000	0.000	0.000	0.000	0.000	0.000	0.000	0.000
22:05 n-6	0.016	0.014	0.011	0.012	0.014	0.012	0.000	0.000	0.000	0.006	0.000	0.004	0.007	0.005	0.007	0.008	0.006	0.006
**22:05 n-3**	0.023	0.020	0.019	0.016	0.016	0.019	0.028	0.034	0.040	0.031	0.028	0.032	0.013	0.008	0.011	0.012	0.011	0.009
**22:06 n-3**	0.081	0.062	0.070	0.064	0.062	0.067	0.108	0.128	0.143	0.169	0.120	0.126	0.202	0.136	0.180	0.206	0.164	0.158
	**0.836**	**0.631**	**0.460**	**0.409**	**0.397**	**0.571**	**0.367**	**0.488**	**0.593**	**0.487**	**0.409**	**0.416**	**0.562**	**0.401**	**0.502**	**0.530**	**0.454**	**0.399**
**w-3**	**0.164**	**0.141**	**0.090**	**0.087**	**0.083**	**0.101**	**0.159**	**0.196**	**0.234**	**0.225**	**0.169**	**0.179**	**0.232**	**0.157**	**0.228**	**0.234**	**0.188**	**0.171**
**T**																		
18:1 t	0.000	0.000	0.000	0.000	0.000	0.000	0.000	0.000	0.000	0.000	0.000	0.000	0.000	0.000	0.000	0.000	0.000	0.000
18:2 t	0.000	0.000	0.000	0.000	0.000	0.000	0.000	0.000	0.000	0.000	0.000	0.000	0.000	0.000	0.000	0.000	0.000	0.000
	**0.000**	**0.000**	**0.000**	**0.000**	**0.000**	**0.000**	**0.000**	**0.000**	**0.000**	**0.000**	**0.000**	**0.000**	**0.000**	**0.000**	**0.000**	**0.000**	**0.000**	**0.000**

Supplemental Figure S3. Results of the analysis of fatty acids composition in the gastrocnemius muscle used in the Supplemental Table 1 of this study. **MO-C:** Mineral oil supplemented group; **MO-HS:** Mineral oil supplemented and hindlimb suspension group; **EPA-C:** High eicosapentaenoic acid fish oil supplemented group; **EPA-HS:** High eicosapentaenoic acid fish oil supplemented and hindlimb suspension group; **DHA-C:** High docosahexaenoic acid fish oil supplemented group; **DHA-HS:** High docosahexaenoic acid fish oil supplemented and hindlimb suspension group**.**

Supplemental Figure S4

p-IRE1

MO-C	MO-HS	EPA-C	EPA-HS	DHA-C	DHA-HS
0.6153585	0.5497207	0.4044116	0.7700508	1.630951	1.999162
0.8997738	1.164103	1.014536	*1.857821*	2.355515	1.460809
0.9194162	*2.687632*	*3.556192*	0.7280849	0.8740129	*5.567465*
1.565451	1.006157	1.698187	0.9563621	1.482459	2.452181
0.2083717	0.4683085	0.390644	1.048939	0.7234957	0.6945592
0.9089412	0.5989404	0.7149263	0.4225054	1.034033	0.9780508
1.557801	0.7225244	1.363589	0.9749871	1.166191	0.8963407
1.324886	0.7502297	1.619482	1.015114	1.070303	1.51302

p-JNK1

MO-C	MO-HS	EPA-C	EPA-HS	DHA-C	DHA-HS
0.3871916	0.3975725	0.4863653	0.4059652	0.5834132	0.3501655
0.4874083	0.2735124	0.8336885	0.6581348	0.6120651	1.243006
1.624972	1.25753	*2.101955*	0.9493784	1.18002	1.450953
1.500428	0.7043236	0.4065093	0.4190323	1.033029	0.6854423
0.3809046	0.8976814	0.8587769	0.3268104	0.2172374	0.2996055
1.180888	1.595899	0.9892865	1.006252	1.553147	0.6189446
1.645584	0.4753423	0.1841554	1.05153	0.5587942	1.506965
0.7926243	1.194505	0.7764287	1.758858	0.6268156	1.316148

Total JNK1

MO-C	MO-HS	EPA-C	EPA-HS	DHA-C	DHA-HS
0.7023358	0.7807512	1.126911	1.489938	1.576421	1.182366
1.706282	1.268036	1.684552	1.260394	1.379447	1.035032
0.6138797	0.9206573	1.157587	0.9704418	1.127453	1.07681
0.9775026	1.011782	1.209735	0.9547787	1.279452	0.8558885
1.357799	1.310717	1.44964	1.136579	1.228455	0.980612
0.9753177	0.9942121	1.022756	0.7702624	0.6786119	0.6312598
0.8130485	0.5443257	0.54624	1.479133	1.327633	1.106339
0.8538344	0.8728133	1.030575	0.8667182	0.9188889	1.070962

p-JNK1/total JNK1

MO-C	MO-HS	EPA-C	EPA-HS	DHA-C	DHA-HS
0.439367	0.4058354	0.3439688	0.2171535	0.2949514	0.2360302
0.2276609	0.1719062	0.394426	0.4161545	0.3536217	0.9571183
2.109643	1.088596	1.447158	0.7796795	0.8341371	1.073892
1.223329	0.5547938	0.2678099	0.3497768	0.6434799	0.6382633
0.2525267	0.6165098	0.5332699	0.258835	0.159185	0.2750295
1.089907	1.444951	0.8707169	1.175966	*2.060241*	0.8826133
1.821924	0.786094	0.3034784	0.6399434	0.3788791	1.226145
0.8356426	1.231951	0.6781856	1.826753	0.6140499	1.10626

p-p38

MO-C	MO-HS	EPA-C	EPA-HS	DHA-C	DHA-HS
0.7290294	0.3726717	1.368305	0.09549447	0.1567636	0.4370244
0.8461534	0.551604	0.31547	0.5616307	1.199858	0.603361
0	0.2226684	0.05338126	0.1117527	0.1276871	0.7128024
1.424817	0.3007653	0.6838436	1.012428	0.6256877	1.744648
0	0.4798277	1.046334	0.6451332	0.1218611	1.359512
1.114661	0.6737167	0.4442618	0.6915135	1.611775	0.04808569
0.8575584	0.2499453	1.011951	0.8550895	1.113358	0.7644187
1.027781	0.7969056	1.15758	*2.215019*	0.1058038	1.038571

Total p38

MO-C	MO-HS	EPA-C	EPA-HS	DHA-C	DHA-HS
0.9365178	0.6888247	0.7310548	0.7069595	1.184724	1.065191
0.3101498	0.1740624	0.7106556	0.5097835	0.7819099	0.569061
0.6018504	0.6719724	0.7864144	0.3008575	0.3931499	1.115617
2.151482	0.71633	1.266323	1.231333	1.116805	1.179705
1.271954	0.6087031	1.358421	1.720866	1.389281	0.3969254
1.276328	0.5658719	0.6607621	0.5914145	1.477918	0.8968603
0.7052578	0.8355139	0.916728	0.3089169	1.040816	1.017373
0.7464595	0.4569152	1.44857	0.6780984	0.1911304	0.3776686

p-p38/total p38

MO-C	MO-HS	EPA-C	EPA-HS	DHA-C	DHA-HS
0.5601808	0.3893292	1.34689	0.09720374	0.09521984	0.2952415
1.963256	2.280456	0.3194465	0.7928013	1.104263	0.762988
0	0.238455	0.04884686	0.2672984	0.2337159	0.4597833
0.4765634	0.302144	0.3886077	0.5916811	0.4031619	1.064225
0	0.6822644	0.666667	0.3244705	0.07591854	2.96447
0.7558805	1.030462	0.5819246	1.012002	0.9439021	0.04640491
1.052419	0.2589191	0.9554148	2.395757	0.9258346	0.650315
1.191701	1.509538	0.6916466	2.827207	0.47912	2.380115

BiP

MO-C	MO-HS	EPA-C	EPA-HS	DHA-C	DHA-HS
0	0.2807142	1.065777	1.357345	0.8471891	0.2648359
0.8775132	0.2957402	0.2632837	0.2643753	0.4639175	0.1775585
1.153148	0.4495475	0.9144222	0.3425869	0.4699619	0.3920566
0.9693384	0.2669658	0.7816318	0.2923143	0.6335202	0.6537158
0.9757515	0.9718959	1.811129	1.52732	1.313757	0.9616829
1.101882	0.3417288	0.8086839	0.4733619	0.583824	0.8605221
1.147827	1.171393	0.9317048	0.8206658	1.086263	0.8239077
0.7745399	0.487431	1.064199	0.3442866	0.7676737	0.2835129

PDI

MO-C	MO-HS	EPA-C	EPA-HS	DHA-C	DHA-HS
0.5315657	0.2042233	0.50086	0.5072151	0.573514	0.3573191
1.23878	1.05621	0.7158358	0.965629	0.6405954	0.4574019
1.778726	1.496685	1.02199	0.3641724	0.596355	0.505756
0.4509286	0.414589	0.5634969	0.3337305	0.4737948	0.4734475
1.070917	0.8171548	0.9285584	0.9124311	1.114486	1.289647
1.11959	0.9437391	1.259529	1.034163	1.514888	2.060867
0.9281873	1.726753	1.950918	1.54825	2.292979	3.048619
0.8813055	0.8296435	0.9381829	0.8741558	1.10552	1.100499

CHOP

MO-C	MO-HS	EPA-C	EPA-HS	DHA-C	DHA-HS
0.4054731	0.796629	1.319978	1.741857	1.872081	4.209004
2.03895	2.229763	1.574215	2.273082	2.152474	0.2562622
1.321636	2.180821	2.257548	2.222811	2.935706	2.720378
0.2339406	0.3375282	0.9856287	1.187397	2.77637	2.846222
0.8396529	0.7395914	0.709124	0.6809554	0.4247961	0.5880466
1.282733	1.169081	2.049561	1.155932	1.026089	0.891471
0.7202581	0.7898347	0.7333388	0.5047874	0.5038145	0.1126229
1.157356	0.4675514	0.4577951	0.2489748	0.268135	0.3043923

Caspse 3

MO-C	MO-HS	EPA-C	EPA-HS	DHA-C	DHA-HS
0.8130626	1.110164	1.02688	1.76136	0.9353209	0.9968339
1.342096	1.580731	1.133577	1.584083	0.6919156	0.8467507
0.8448417	1.613304	1.359702	2.266006	2.427004	1.853052
0	*3.092324*	1.182628	1.019917	1.080899	2.841983
0.9440381	1.35222	1.284026	1.480267	0.9387398	0.8509735
0.8446205	1.180406	0.909142	1.072325	1.151763	1.748046
1.010927	1.577311	1.56993	2.006764	1.814891	2.278161
1.200414	1.408836	1.216581	1.194373	1.151727	1.620704

Supplemental Figure S4. Results of the quantitative analysis of western blot assays used in the Figures 1-4 of this study. **MO-C:** Mineral oil supplemented group; **MO-HS:** Mineral oil supplemented and hindlimb suspension group; **EPA-C:** High eicosapentaenoic acid fish oil supplemented group; **EPA-HS:** High eicosapentaenoic acid fish oil supplemented and hindlimb suspension group; **DHA-C:** High docosahexaenoic acid fish oil supplemented group; **DHA-HS:** High docosahexaenoic acid fish oil supplemented and hindlimb suspension group**.** Grubb’s test was used to exclude outliers (blue numbers).

Supplemental Figure S5

BiP

MO-C	MO-HS	EPA-C	EPA-HS	DHA-C	DHA-HS
0.6757534	0.5694513	0.9928343	0.2054358	0.4982093	0.3826492
0.4247056	0.5221807	0.6894848	0.2418308	0.7078679	0.708095
1.089224	0.2590941	1.911067	0.319735	1.074549	0.2052097
0.5166567	0.4915888	0.5461434	0.2666981	0.5829486	0.3818604
0.9318824	0.3833502	1.235712	0.3624342	0.3421474	0.2354435
1.401891	0.390218	0.6872534	0.2030624	*1.681313*	0.1557699
0.9047857	0.4948895	2.013075	0.5426381	0.7209339	0.2864181
0.9242067		0.6383536		0.4627544	0.2282302
2.227358		0.6398183		0.9528923	0.8931368
2.544628		0.4627929		0.8575469	

CHOP

MO-C	MO-HS	EPA-C	EPA-HS	DHA-C	DHA-HS
0.902231	1.128686	0.8625171	0.1564485	1.095678	1.03605
0.6248297	1.337576	1.206302	0.8461994	0.5817826	2.127287
0.7957022	0.978435	1.258215	0.5111961	1.338606	0.2977495
0.8090371	1.267973	0.03885789	0.392368	1.282039	0.8815518
0.8095656	0.6171679	0.9153166	0.9745384	0.5624084	1.454429
1.314373	1.101411	0.7932865	0.5498063	1.785822	0.4276458
0.9218782	0.3396636	2.065371	1.010495	0.630662	0.4840388
1.023341		0.9456825		0.8921255	0.7348703
1.497269		0.8542523		0.6273685	1.733819
1.833315		1.759833		0.9561359	

IRE1

MO-C	MO-HS	EPA-C	EPA-HS	DHA-C	DHA-HS
0.4732153	2.738991	0.5454901	0.3985299	0.4063582	0.7120716
1.087138	1.691873	0.6875758	0.7621092	0.8690747	1.969753
0.4115959	0.8997199	1.042739	1.214994	0.716844	0.4668953
0.7543353	3.297846	0.3128085	1.034748	0.7670756	1.502242
1.105133	1.285861	0.5290012	1.339589	*1.838705*	1.47371
0.8665618	1.147387	0.9558883	1.046807	*4.157131*	0.5409204
0.5554188	0.8017245	1.003751	*4.904351*	0.8490603	1.158449
1.182867		0.9648842		0.6946309	0.576168
2.397172		0.8188858		0.6908216	*5.216033*
4.150988		*9.090999*		0.3800569	

PERK

MO-C	MO-HS	EPA-C	EPA-HS	DHA-C	DHA-HS
0.5047247	1.598417	0.6322753	0.7452311	0.504814	0.9815231
1.059611	1.755183	0.8781813	0.6741164	0.7323231	0.9733393
0.4770791	0.7425437	1.200286	0.9753208	0.8601922	0.5881153
0.7052848	2.557127	0.7201411	0.7281357	0.7171966	0.6830757
0.937714	0.9564323	0.4551275	0.8980023	0.4971283	0.8903552
0.8387859	1.302562	0.5277454	0.7894914	*3.996087*	0.4312177
0.5229149	0.855108	0.7363181	*5.087871*	1.033071	0.760592
1.046293		0.8653933		0.5348916	0.7513523
2.048167		1.060492		0.476116	*5.641008*
6.304817		*6.715239*		0.6817372	

BECLIN

MO-C	MO-HS	EPA-C	EPA-HS	DHA-C	DHA-HS
0.8739173	0.8880078	0.2775126	*0.6496614*	0.7050629	0.4219354
0.6486602	0.4455382	0.803623	0.3063111	0.9218169	0.4941479
0.4073386	0.5405679	0.3699439	0.2545373	0.7293742	0.2617336
0.6454055	*1.861575*	0.4532414	0.3595537	0.4451738	0.7695662
0.9788908	0.1904831	0.4987341	0.3968844	0.4978179	0.770813
0.8457919	0.3306444	0.312064	0.32556	*3.946539*	0.4001155
1.146033	0.2216228	0.9204435	*2.280045*	0.426004	0.1669161
1.178774		0.5717387		0.4350697	0.2660117
2.022772		0.8160527		0.9469664	*1.714696*
2.965868		*3.835564*		0.9587905	

LC3II

MO-C	MO-HS	EPA-C	EPA-HS	DHA-C	DHA-HS
1.116179	0.7964437	0.8788111	0.5628256	0.6919904	1.336161
1.4325	1.666269	1.187223	2.213101	1.608317	1.439946
0.6229984	1.261857	1.88999	1.877677	1.53446	1.221938
1.165766	0.7843252	0.3466019	1.130749	1.315344	1.664533
1.126791	1.803419	1.056537	2.203491	1.76136	3.452049
1.080257	2.542684	1.583277	2.134644	0.8727183	2.481975
1.188917	0.8231221	1.477749	0.4389298	0.8133448	3.227003
1.495147		1.511968		1.368237	1.051582
0.8641379		1.78972		1.234892	0.08082543
0.4605579		1.001692		0.9345136	

ATG14

MO-C	MO-HS	EPA-C	EPA-HS	DHA-C	DHA-HS
0.4709248	2.292059	0.6871157	1.700265	0.4394661	0.9747432
1.143562	2.623733	0.7698187	0.8956889	0.6785618	1.475307
0.467261	1.569764	0.922345	0.675463	0.9092388	0.5374382
0.8271827	2.402479	0.9502319	0.6746818	0.9268711	2.602895
1.122893	2.035996	0.4519837	1.296652	1.095576	2.717826
0.6445547	2.315546	0.7259358	1.393773	*2.426014*	1.501587
0.6305348	2.076535	0.4170814	2.40671	0.9246241	1.735312
1.113644		0.8475837		0.8451729	0.5538478
1.897807		1.038668		0.5622895	3.520921
4.981066		*5.0892*		0.5853159	

Supplemental Figure S5. RT-PCR results after $2^{-\Delta\Delta CT}$ calculation for the relative expression of genes used in the Figures 5 and 6 of this study. **MO-C:** Mineral oil supplemented group; **MO-HS:** Mineral oil supplemented and hindlimb suspension group; **EPA-C:** High eicosapentaenoic acid fish oil supplemented group; **EPA-HS:** High eicosapentaenoic acid fish oil supplemented and hindlimb suspension group; **DHA-C:** High docosahexaenoic acid fish oil supplemented group; **DHA-HS:** High docosahexaenoic acid fish oil supplemented and hindlimb suspension group**.** Grubb’s test was used to exclude outliers (blue numbers).
